# During hormone depletion or tamoxifen treatment of breast cancer cells the estrogen receptor apoprotein supports cell cycling through the retinoic acid receptor α1 apoprotein

**DOI:** 10.1186/bcr2827

**Published:** 2011-02-07

**Authors:** Marcela D Salazar, Maya Ratnam, Mugdha Patki, Ivana Kisovic, Robert Trumbly, Mohamed Iman, Manohar Ratnam

**Affiliations:** 1 Department of Biochemistry and Cancer Biology, Medical University of Ohio, 3000 Arlington Avenue, Toledo, OH 43614, USA; 2 Department of Medicine, Medical University of Ohio, 3000 Arlington Avenue, Toledo, OH 43614, USA; 3 Bioinformatics & Proteomics/Genomics Core Division, Medical University of Ohio, 3000 Arlington Avenue, Toledo, OH 43614, USA

## Abstract

**Introduction:**

Current hormonal adjuvant therapies for breast cancer including tamoxifen treatment and estrogen depletion are overall tumoristatic and are severely limited by the frequent recurrence of the tumors. Regardless of the resistance mechanism, development and progression of the resistant tumors requires the persistence of a basal level of cycling cells during the treatment for which the underlying causes are unclear.

**Methods:**

In estrogen-sensitive breast cancer cells the effects of hormone depletion and treatment with estrogen, tamoxifen, all-*trans *retinoic acid (ATRA), fulvestrant, estrogen receptor α (ER) siRNA or retinoic acid receptor α (RARα) siRNA were studied by examining cell growth and cycling, apoptosis, various mRNA and protein expression levels, mRNA profiles and known chromatin associations of RAR. RARα subtype expression was also examined in breast cancer cell lines and tumors by competitive PCR.

**Results:**

Basal proliferation persisted in estrogen-sensitive breast cancer cells grown in hormone depleted conditioned media without or with 4-hydroxytamoxifen (OH-Tam). Downregulating ER using either siRNA or fulvestrant inhibited basal proliferation by promoting cell cycle arrest, without enrichment for ErbB2/3+ overexpressing cells. The basal expression of RARα1, the only RARα isoform that was expressed in breast cancer cell lines and in most breast tumors, was supported by apo-ER but was unaffected by OH-Tam; RAR-β and -γ were not regulated by apo-ER. Depleting basal RARα1 reproduced the antiproliferative effect of depleting ER whereas its restoration in the ER depleted cells partially rescued the basal cycling. The overlapping tamoxifen-insensitive gene regulation by apo-ER and apo-RARα1 comprised activation of mainly genes promoting cell cycle and mitosis and suppression of genes involved in growth inhibition; these target genes were generally insensitive to ATRA but were enriched in RAR binding sites in associated chromatin regions.

**Conclusions:**

In hormone-sensitive breast cancer, ER can support a basal fraction of S-phase cells (i) without obvious association with ErbB2/3 expression, (ii) by mechanisms unaffected by hormone depletion or OH-Tam and (iii) through maintenance of the basal expression of apo-RARα1 to regulate a set of ATRA-insensitive genes. Since isoform 1 of RARα is genetically redundant, its targeted inactivation or downregulation should be further investigated as a potential means of enhancing hormonal adjuvant therapy.

## Introduction

Most breast tumors in both premenopausal and postmenopausal women express estrogen receptor type alpha (ER). Tamoxifen is a Selective Estrogen Receptor Modulator (SERM) widely used for adjuvant therapy in the treatment of ER+ breast cancer. In the hormone-sensitive tumors, tamoxifen acts as a partial antagonist, impairing ER function by competing with estrogen for binding to the receptor [1]; however, more than three years of tamoxifen treatment only results in approximately 50% reduction in the incidence of invasive breast cancer in women at high risk, whereas about a third of ER+ breast tumors are intrinsically resistant to tamoxifen [2,3].

Third generation aromatase inhibitors (AI) present a valuable alternative to tamoxifen adjuvant therapy in postmenopausal women with ER+ breast cancer [4-6]. Aromatase activity is essential for catalyzing the conversion to estrogen of steroid precursors in peripheral tissues, the major source of estrogen production in postmenopausal women. Upon treatment with AI, aromatase activity is reduced by at least 96% and circulating estrogen is virtually absent, inhibiting hormone-dependent tumor growth [7]. In spite of the sensitivity of tamoxifen-resistant tumors to AI, breast tumors also acquire resistance to AI after long term treatment, resulting in disease recurrence and aggressive tumor growth [8,9]. Clinical trials are underway to assess the possibility of delaying the onset of resistance by administering AI for two to three years following two to three years of tamoxifen treatment [10,11]. The mechanistic basis underlying breast tumor resistance to either hormone depletion or to tamoxifen is still inadequately understood. In the vast majority of cases, resistance must occur through hormone-independent ER signaling events [12,13]. Accordingly, Selective Estrogen Receptor Downregulators (SERDs, for example, Faslodex) have been found to be effective inhibitors of ER+ breast tumor growth but their utility is limited to their use as second or third line therapeutics in postmenopausal women with metastatic disease due to their broader impact on physiological ER signaling pathways in normal tissues [14,15]. Therefore, it is imperative to continue to identify critical downstream events of ER signaling in breast cancer.

Breast cancer therapy trials have also been designed to explore the effect of retinoid compounds either alone or in combination with tamoxifen [16]. In *in vitro *and pre-clinical models of breast cancer using MCF-7 cell xenografts, *all-trans*- retinoic acid (ATRA) alone or in combination with tamoxifen induced cell cycle arrest and apoptosis, leading to tumor regression through activation of multiple signal transduction pathways [17-19]. Synergistic anti-tumor effects have been noted *in vitro *for the combination of retinoid and tamoxifen and multiple molecular mechanisms for the ligand effects have been reported [20,21]. However, toxicity issues due to ATRA treatment was a challenge in patients with advanced breast cancer during phaseI/II clinical trials [22]. Fenretinide, a synthetic amide of retinoic acid, has a better toxicological profile acting on both ER+ and ER- breast tumors principally by inducing apoptosis by both retinoic acid receptor (RAR) -dependent and -independent mechanisms; this drug showed a modest chemopreventive effect only in younger premenopausal women [23].

Hormonal adjuvant therapy of breast cancer is overall tumoristatic with cell death balancing a basal level of cell proliferation [24]. From a fundamental mechanistic standpoint, for resistance to develop in the long term during either hormone depletion or tamoxifen adjuvant therapy, the latent tumors must sustain a basal level of cell cycling to enable the generation and/or progression of genetic or epigenetic changes [25] leading to resistance. It is the premise of this study that understanding the mechanisms that support the persistence of a small fraction of cells in S-phase throughout the course of hormonal adjuvant therapy in breast cancer will shed light on this critical precondition for the eventual development of resistance to the treatments. Since estrogen-independent ER signaling has been implicated in the development of resistance to adjuvant therapy, it was the goal of this study to examine the relationship between hormone-independent actions of ER and the basal cycling state of estrogen deprived breast cancer cells. Further, the ER-RAR axis has only been investigated in the context of ligand-dependent effects [26]; it was therefore of additional interest to explore a possible interplay between the apo- forms of ER and RAR and its impact on basal proliferation, that is, under conditions of hormone depletion or tamoxifen antagonism.

Estrogen-sensitive breast cancer cell lines (MCF-7, T47 D and ZR-75-1) have proven to be exceptionally reliable predictive models both *in vitro *and *in vivo *for clinical drug response and the development of clinical drug resistance in breast cancer [27-30]. We have observed that the expected basal proliferating state of hormone-depleted or tamoxifen treated breast cancer cells may be reproduced *in vitro *in established cell lines for an indefinite period by avoiding the common practice of intermittently replenishing the culture media, thus avoiding depletion of autocrine growth factors. We, therefore, used *in vitro *models to investigate the potential impact of hormone-independent actions of ER on the survival or proliferation of hormone-sensitive breast cancer cells and the related mechanisms under conditions that mimic hormonal adjuvant therapy, that is, estrogen-depletion and tamoxifen treatment.

## Materials and methods

### Chemicals and reagents

Dulbecco's minimum essential medium (DMEM), glutamine and penicillin/streptomycin/glutamine stock mix were purchased from Life Technologies, Inc. (Carlsbad, CA, USA). Fetal bovine serum (FBS) and charcoal-stripped FBS were from Invitrogen (Carlsbad, CA, USA). Fugene 6 and Dharmafect 1 were from Roche Diagnostics (Indianapolis, IN, USA) and Dharmacon (Thermo Scientific Dharmacon, Inc., Lafayette, CO, USA), respectively. ERα (J-003401-12), RARα (J-003437-07) and control (D-001810-02) small interfering RNA (siRNA) were purchased from Dharmacon (Thermo Scientific Dharmacon, Inc.). Affinity purified rabbit and mouse antibodies to human ERα (sc-543), RARα (sc-551), RARβ (sc-552), RARγ (sc-550) and glyceraldehyde-3-phosphate dehydrogenase (GAPDH; sc-47724) were from Santa Cruz Biotechnologies (Santa Cruz, CA, USA). Peroxidase-conjugated secondary antibody was from Vector Laboratories (Burlingame, CA, USA). For standard PCR, HotStart Taq Plus DNA Polymerase was used (Qiagen, Germantown, MD, USA). Reagents for real time PCR, primers and TaqMan probes were purchased from Applied Biosystems (Branchburg, NJ, USA). PI/RNase staining buffer was from BD Pharmigen (San Diego, CA, USA). The Guava Nexin Reagent was purchased from Guava Technologies (Guava Technologies, Inc., Hayward, CA, USA). The protease inhibitor cocktail kit was obtained from Pierce Biotechnology (Thermo Scientific, Rockford, IL, USA). 17β-estradiol (E_2_), 4-hydroxytamoxifen (OH-Tam), all-*trans*-Retinoic acid (ATRA) and fulvestrant were purchased from Sigma Aldrich (Saint Louis, MO USA). ATRA stock solution (5 mmol/L) was made in a mixture of 50% ethanol and 50% DMSO (Fisher Chemical, Fair Lawn, NJ, USA). First strand cDNA from human peripheral blood leukocytes (PBL), thymus and spleen were obtained from Biochain Institute (Biochain Institute Inc., Hayward, CA, USA). Total RNA from normal human breast and human breast tumors were obtained from Biochain Institute Institute and Clonetech (Clonetech Laboratories Inc., Mountain View, CA, USA).

### Cell culture and treatment with fulvestrant or ATRA

MCF-7 and T47 D (American Type Culture Collection) cells were cultured in DMEM supplemented with FBS (10%), penicillin (100 unit/ml), streptomycin (100 μg/ml) and L-glutamine (2 mM). ZR-75-1 (American Type Culture Collection) cells were cultured in RPMI 1640 supplemented with FBS (10%), penicillin (100 unit/ml), streptomycin (100 μg/ml) and L-glutamine (2 mM). Hormone depleted cells were grown in low glucose phenol-red free media supplemented with 5% charcoal-stripped FBS (v/v) and L-glutamine (2 mM) for 48 hours before the experiments. Hormone-depleted MCF-7 cells were grown to 60 to 70% confluency in six-well plates and treated with vehicle or ATRA (1 μM) for 24 hours. After 24 hours, the cells were harvested for total RNA isolation and mRNA profiling. Hormone-depleted MCF-7 cells were grown to 30 to 40% confluency in six-well plates and treated with vehicle or fulvestrant (100 nM) for up to 4 days. After 72 hours to 96 hours, the cells were harvested for isolation of total RNA and protein.

### Transfection and gene silencing

Cells were plated at 20% confluence in low glucose phenol red free medium supplemented with 5% charcoal stripped FBS and glutamine 24 hours to 48 hours prior to transfection. Treatment with vehicle (ethanol), E_2 _(1 nM) or OH-Tam (100 nM) was begun an additional 24 hours later. Cells were transfected with control siRNA, ERα siRNA or RARα siRNA (100 pmol/mL) in 24-well microplates or 25 cm^2 ^flasks using 2 μl and 12.5 μl of Dharmafect 1 (Thermo Scientific Dharmacon Inc.), respectively, according to the vendor's protocol. The cell culture medium was not replenished for the duration of the experiment. In the RARα1 rescue experiments, 2 × 10^6 ^cells were co-transfected with 2 μg of either the vector plasmid or RARα1 expression plasmid and with control siRNA or ERα siRNA by nucleofection using the Kit V Amaxa Nucleofection System (Amaxa Biosystems, Allendale, NJ, USA) according to the vendor's instructions. In the RARα1 rescue of fulvestrant- treated cells and RARα1 overexpression experiments, 2 μg RARα1 expression plasmid was introduced at a cell density of 2.5 × 10^5 ^cells per well in six-well plates using Fugene 6 according to the manufacturer's protocol.

### Cell growth assay

Cells were seeded in 24-well microplates at 20% confluence in phenol-red free media supplemented with 5% charcoal-stripped FBS (v/v) and incubated at 37°C with 5% CO_2 _for 24 h. Cells were transfected with either control siRNA or siRNA targeting ERα using Dharmafect 1. Twenty-four hours after transfection the media was replaced with fresh phenol-red free media supplemented with 5% charcoal-stripped FBS (v/v) and the cells were treated with vehicle (ethanol), E_2 _(1 nM) or OH-Tam (100 nM) for the following five days; the culture media was not changed during this period but E_2 _and OH-Tam were replenished every 48 h. Viable cell counts were monitored using the trypan blue dye exclusion assay at intervals of 24 h.

### Cell cycle analysis

Cells were trypsinized and harvested in phenol red free medium supplemented with charcoal stripped FBS. Cells (1 × 10^6^) were washed and resuspended in 500 μl PBS. The cells were fixed by adding 500 μl 100% ice cold ethanol, drop-wise with agitation and incubated on ice for 20 minutes. The cells were sedimented by brief centrifugation at 200 xg for five minutes and the excess ethanol decanted. After the remaining ethanol was dried off, the cells were resuspended in 500 μl of PI/RNase solution. The cells were incubated in the dark at room temperature for 20 minutes and the cell cycle distribution determined by flow cytometric analysis using a FACSCalibur cell analyzer (BD Biosciences, San Jose, CA, USA). The data were acquired with BD CellQuest Pro software and analyzed using ModFit LT software.

### Apoptosis assay

Early stage apoptosis of cells was measured by Guava Nexin analysis using the Guava Nexin Reagent staining kit according to the manufacturer's instructions. Briefly, 8 × 10^4 ^cells were incubated for 20 minutes at room temperature with the Guava Nexin Reagent and 2,000 cells per sample were analyzed using the Guava System.

### Western blot

Cells were harvested by trypsinization, lysed in a high salt-detergent buffer (400 nM NaCl; 10 nM Tris, pH 8.0; 1 mM EDTA; 1 mM EGTA; β-mercaptoethanol; and 0.1% Triton x-100) containing a protease inhibitor cocktail kit and incubated on ice for 30 minutes. Cell lysates were heated to 95°C for five minutes. Protein samples (10 to 20 μg) were resolved by electrophoresis on 8% sodium dodecylsulfate-polyacrylamide gels and electrophoretically transferred to PVDF membranes (Millipore Corporation, Bedford, MA, USA). The blots were probed with the appropriate primary antibody and the appropriate horseradish peroxidase conjugated secondary antibody and the protein bands visualized using enhanced chemiluminescence as described [31]. The chemiluminescent signals were quantified using the FluorChem HD2 imaging system (Alpha Innotech/Cell Biosciences, Inc., Santa Clara, CA, USA) and normalized to GAPDH.

### RNA isolation, reverse transcription PCR and Real time PCR

Total RNA from cells was isolated using the RNeasy mini kit (Qiagen, Georgetown, MD, USA). Reverse transcription PCR reactions were performed using 500 ng of total RNA and the high capacity complementary DNA Archive kit (Applied Biosystems) according to the vendor's protocol. cDNAs of RARα1 and RARα2 were amplified by competitive PCR. The upstream and downstream primers used for amplification of RARα1 and RARα2 were as follows: RARα1, 5'-GCCAGGCGCTCTGACCACTC-3' and 5'-AGCCCTTGCAGCCCTCACAG-3'; RARα2, 5'-ACTCCGCTTTGGAATGGCTCAAAC-3' and 5'-AGCCCTTGCAGCCCTCACAG-3'. The cDNA for the house keeping gene glyceraldehyde-3- phosphate dehydrogenase (GAPDH) was amplified and the primer sequences used were as follows: 5'-TGGTCACCAGGGCTGCTTTT-3' and 5'-GGTGAAGACGCCAGTGGACT-3'. The cycling parameters were: 95°C for 15 minutes; 94°C for 30 sec; 60°C for 30 sec; 72°C for 30 sec and 72°C for 10 minutes. RARα1 and RARα2 cDNAs were amplified in the same reaction, yielding products of 222 bp and 182 bp, respectively. PCR products were separated in ethidium bromide-stained 2% agarose gels by electrophoresis. cDNA was also measured by quantitative real time PCR in the 7500 StepOne Plus Real time PCR System (Applied Biosystems). Primers and TaqMan probes for the human ERα, CCNA, CDKN1, ERBB2, ERBB3, MUC20, LYPD1, RARα and GAPDH genes were obtained from the Applied Biosystems inventory. All samples were measured in triplicate and normalized to GAPDH values.

### mRNA profiling

The Affymetrix chips were purchased from Affymetrix (Santa Clara, CA, USA) DNA microarray analysis using Affymetrix was performed as a full service global gene expression study at the transcriptional profiling core facility of the Cancer Institute of New Jersey. Total RNA samples were used to generate labeled cRNAs, which were hybridized to human U133 Plus2.0 Affymetrix microarrays. The expression data were analysed initially using Affymetrix GeneChip Operating Software to create CEL files. The CEL files were imported into the Bioconductor program affylmGUI [32]. The probe set level intensities were quantified and normalized using robust multiarray averaging and quantile normalization. Differential expression between treatments was determined using the limma linear modeling method, and the significance of differences was ranked by the moderated *t*-statistic. The values for signal intensities were corrected for siRNA transfection efficiencies determined using a Green Fluorescent Protein (GFP) reporter expression plasmid. To identify genes differentially expressed under the different treatments, the fold-changes were calculated by dividing the average signal of the treatment by the control, and genes with a fold-change greater or lesser than a given threshold were chosen. The advantage of this approach is that rejection of many false negatives is avoided, compared to requiring a statistically significant difference in expression, but has the potential drawback of including false positives. When we limited the genes in Tables 1 and 2 to those showing significant differential expression at the *P *= 0.05 level by the linear modeling method, 25/54 genes in Table 1 and 24/68 genes in Table 2 were retained. In the reduced sets of genes, similar percentages of genes showed RARα peaks as in the larger gene set, confirming the generality of our result. The Affymetrix data are deposited in GEO (Accession number: [GEO:GSE26298]).

**Table 1:** Tamoxifen Insensitive genes supported by the Apo-ER -> Apo-RARα Axis

Gene	ATRA effect	RAR^a ^binding <10 kb	Fold change siER	Fold change siRARα1	Gene	ATRA effect	RAR^a ^binding <10 kb	Fold change siER	Fold change siRARα1
*LGALS1*	↓	-	0.31	0.46	CASC5	-	-	0.47	0.51
*METTL7A*	↓	-	0.37	0.53	BIRC5	-	-	0.53	0.48
*CDKN3*	↓	-	0.44	0.60	PTTG1	-	-	0.57	0.54
*XK*	↓	-	0.46	0.54	PLK4	-	-	0.56	0.49
*GHR*	↓	-	0.41	0.57	CENPA	-	-	0.58	0.56
*YPEL1*	↓	-	0.58	0.54	TMSB15A	-	-	0.42	0.41
*SHANK2*	↑	**++**	0.58	0.57	C5	-	-	0.38	0.48
*ENY2*	-	**++**	0.54	0.45	CDC2	-	-	0.56	0.59
*ONECUT2*	-	**++**	0.50	0.58	CCNA2	-	-	0.59	0.52
*HIST1H4C*	-	**++**	0.21	0.31	LOC150759	-	-	0.49	0.59
*NCAPH*	-	**++**	0.47	0.53	NCAPG	-	-	0.50	0.51
*CENPN*	-	**++**	0.56	0.55	CENPM	-	-	0.57	0.50
*UBE2T*	-	**++**	0.53	0.60	FAM64A	-	-	0.55	0.58
*PHF19*	-	**++**	0.50	0.50	MND1	-	-	0.51	0.55
*ZNF367*	-	**++**	0.52	0.51	FGFR1	-	-	0.56	0.53
*SNORA72*	-	**++**	0.43	0.58	HELLS	-	-	0.58	0.55
*KIF23*	-	**+**	0.58	0.56	TNFAIP8L1	-	-	0.47	0.55
*ENAH*	-	**+**	0.54	0.50	OVOS2	-	-	0.40	0.58
*MAD2L1*	-	**+**	0.40	0.55	ZNF141	-	-	0.55	0.52
*SMC4*	-	**+**	0.54	0.60	LOC100129673	-	-	0.39	0.48
*SEC31A*	-	**+**	0.55	0.57	ASPM	-	-	0.45	0.45
*SFPQ*	-	**+**	0.60	0.49	EPHX4	-	-	0.51	0.55
*CENPF*	-	**+**	0.51	0.58	HNRPD	-	-	0.36	0.55
*COBL*	-	-	0.54	0.52	ARPC5L	-	-	0.59	0.52
*PBK*	-	-	0.56	0.56	RGS3	-	-	0.57	0.45
*MLF1IP*	-	-	0.44	0.58	SIPA1L1	-	-	0.55	0.58
*SAMHD1*	-	-	0.60	0.53					

**^a ^**RAR binding site identified within 10 kb of the transcription start site (Hua *et al*., 2009).

**++ **RAR binding site detected under high stringency (Hua *et al*., 2009).

+ RAR binding site detected under low stringency (Hua *et al*., 2009).

↑ Up-regulated by ATRA (1 μM, 24 h) ≥2-fold.

↓ Down-regulated by ATRA (1 μM, 24 h) ≤50%.

Abbreviations: ER, estrogen receptor; RAR, retinoic acid receptor; ATRA, *all *trans-retinoic acid; si, small interfering RNA.

**Table 2:** Tamoxifen Insensitive genes Repressed by the Apo-ER -> Apo-RARα Axis

Gene	ATRA effect	RAR ^a ^binding <10 kb	Fold change siER	Fold change siRARα1	Gene	ATRA effect	RAR ^a ^binding <10 kb	Fold change siER	Fold change siRARα1
*IFI44L*	↓	**+**	1.69	2.22	*UBL3*	-	**++**	1.61	1.58
*IFI44*	↓	-	2.09	2.28	*SLC7A11*	-	**++**	1.71	2.03
*CCPG1*	↓	-	1.67	1.56	*KIAA0652*	-	**++**	1.57	1.63
*SLC25A36*	↓	-	1.66	1.73	*PHLDB1*	-	**++**	1.64	1.55
*SETD5*	↓	**++**	1.52	2.11	*CCNT2*	-	-	1.53	1.63
*MALL*	↓	-	1.67	1.81	*STX3*	-	-	1.52	1.59
*LYPD1*	↓	-	1.66	1.59	*CLN8*	-	-	1.71	1.72
*FAM186A*	↓	-	1.64	1.75	*UBXN10*	-	-	1.73	1.64
*GPR158*	↓	-	1.81	1.54	*PVR*	-	-	1.63	2.39
*CDKN1A*	↓	-	1.70	1.51	*TNFRSF10A*	-	-	1.54	1.62
*CEACAM6*	↑	**+**	2.35	1.63	*FLJ31958*	-	-	1.71	1.52
*LGALS3BP*	↑	-	1.98	1.92	*C18orf25*	-	-	1.50	1.61
*CTSS*	↑	-	1.50	1.86	*TAPBP*	-	-	1.62	1.51
*SELL*	↑	-	1.75	1.71	*ADAM17*	-	-	1.58	1.63
*SHROOM1*	↑	-	1.68	1.59	*HCP5*	-	-	1.59	2.52
*CP*	↑	**++**	1.71	1.75	*DISC1*	-	-	1.80	1.74
*ABHD2*	↑	**++**	1.56	1.57	*SP100*	-	-	1.77	1.61
*ABLIM1*	↑	**++**	1.56	1.55	*AASS*	-	-	1.53	2.17
*ALOX5*	↑	-	1.72	2.09	*HLA-G*	-	-	1.50	2.33
*HLA-C*	↑	-	1.51	2.51	*HLA-B*	-	-	1.61	2.08
*MAP1B*	↑	-	2.12	1.51	*RSAD2*	-	-	1.84	3.19
*ARHGAP1*	↑	-	1.58	1.55	*SGSM3*	-	-	1.72	1.50
*SP110*	↑	-	1.58	1.63	*HLA-J*	-	-	1.52	2.04
*RTP4*	↑	-	1.60	1.81	*SESN1*	-	-	1.53	1.60
*MUC20*	↑	-	1.75	1.91	*ANO10*	-	-	1.51	1.64
*RNF38*	-	**+**	1.80	1.69	*SAMD9*	-	-	1.68	1.56
*C9orf80*	-	**+**	1.64	1.53	*FLJ13197*	-	-	1.68	1.57
*CEACAM5*	-	-	2.35	1.70	*PGLS*	-	-	1.54	1.81
*CNOT4*	-	-	1.79	1.92	*LMO3*	-	-	1.61	1.58
*PLXNA2*	-	-	1.65	1.86	*DNAJC21*	-	-	1.68	1.61
*TTC9*	-	-	1.76	1.51	*SETD2*	-	-	1.52	2.11
*EIF5A2*	-	-	1.90	1.67	*ZNF24*	-	-	2.10	1.68
*FLCN*	-	**++**	1.51	1.53	*TTLL11*	-	-	1.72	1.51
*BAZ2A*	-	**++**	1.53	1.58	*ZNF544*	-	-	1.58	1.71

**^a ^**RAR binding site identified within 10 kb of the transcription start site (Hua *et al*., 2009).

**++ **RAR binding site detected under high stringency (Hua *et al*., 2009).

+ RAR binding site detected under low stringency (Hua *et al*., 2009).

↑ Up-regulated by ATRA (1 μM, 24 h) ≥2-fold.

↓ Down-regulated by ATRA (1 μM, 24 h) ≤50%.

Abbreviations: ER, estrogen receptor; RAR, retinoic acid receptor; ATRA, *all *trans-retinoic acid; si, small interfering RNA.

### Statistical analyses

Experimental values are presented as mean ± standard deviation (s.d.). The statistical significance of differences (*P*-value) between values being compared was determined using analysis of variance. In all cases, the differences noted in the text are reflected by a *P*-value of <0.001.

## Results

### In estrogen-sensitive MCF-7 cells basal proliferation is supported by ER in the absence of hormone

**Figure 1:**
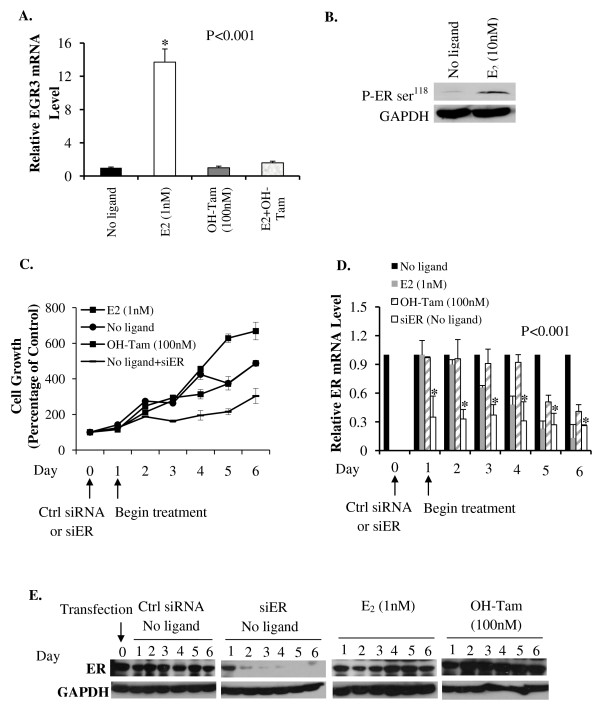
**Hormone-independent effects of ER on the proliferation of hormone-sensitive MCF-7 cells**. In all the experiments, MCF-7 cells were first cultivated in phenol red free DMEM containing 5% charcoal-stripped FBS for 48 hours to deplete hormone. EGR3 mRNA levels in the cells were measured by real time RT-PCR after a brief (8 h) treatment with vehicle (No ligand), E_2 _(1 nM) or OH-Tam (100 nM) **(A)**. Cells were also treated with vehicle (No ligand) or E_2 _(10 nM) for 30 minutes and ligand-dependent activation of ER was analyzed by western blot using a specific antibody to detect phosphorylation at ser^118 ^of ER; GAPDH was probed as a loading control **(B)**. Hormone-depleted MCF-7 cells were transfected with either ER siRNA or control siRNA and maintained in hormone-depleted conditioned media without further replenishment of the media. Twenty-four hours after transfection, cells were treated with vehicle (No ligand), E_2 _(1 nM) or OH-Tam (100 nM); the treatments were repeated every 48 hours without changing the media. Following the treatment, viable cells were counted daily for six days by the Trypan Blue dye exclusion assay **(C)**. In parallel, cells were harvested on each day of the treatments and total RNA extracted from them; the mRNA for ER was measured by real time RT-PCR and the values were normalized to those for GAPDH **(D)**. In addition, cells were harvested in parallel on each day of the treatments for western blot analysis using antibody to ER; GAPDH was probed in each blot as a loading control **(E)**. *P*-values for the differences noted in the text were ≤0.001.

In the following studies, in experiments in which MCF-7 cells were depleted of hormone, the virtual absence of hormone was confirmed in two ways. First, the effect of OH-Tam on the expression of the classical E_2 _target gene, EGR3 (Early Growth Response 3 gene) was examined as a functional test. The expression of EGR3 mRNA is exquisitely sensitive to upregulation by E_2, _in a manner that is completely antagonized by OH-Tam. In the hormone depleted cells, the inability of OH-Tam to further decrease EGR3 mRNA indicated the virtual absence of hormone (Figure 1A). Second, the effect of E_2 _on the relative phosphorylation level at ser^118 ^of ER was examined. The binding of E_2 _strongly induces phosphorylation of ER at this site [33]. The absence of hormone was further confirmed by the observation of much lower phosphorylation at ser^118 ^of ER in the hormone depleted cells, when compared with control cells treated with E_2 _(Figure 1B).

When hormone depleted MCF-7 cells were seeded at a low confluency (<20 percent) and grown, in the absence of hormone and without replenishing the medium, they continued to proliferate and the proliferation was not inhibited by OH-Tam; we will call this 'basal' proliferation. Under these conditions, we have monitored basal proliferation in MCF-7 cells for more than three months of continuous culture. Treatment with E_2 _stimulated the cell growth demonstrating that the cells were hormone-sensitive (Figure 1C). The basal proliferation was diminished by knocking down ER (Figure 1C). The expression level of ER mRNA (Figure 1D) progressively decreased with ligand (E_2 _or OH-Tam) treatment but the ER protein level (Figure 1E) was stabilized by the ligands during the treatment period; however, ER siRNA specifically and substantially down-regulated ER mRNA (within 24 h) and ER protein (within 48 h) (Figure 1D and 1E). The results demonstrate a profound role for apo-ER in supporting basal proliferation in hormone-sensitive MCF-7 cells.

### Apo-ER supports MCF-7 cell proliferation by promoting cell cycle progression

**Figure 2:**
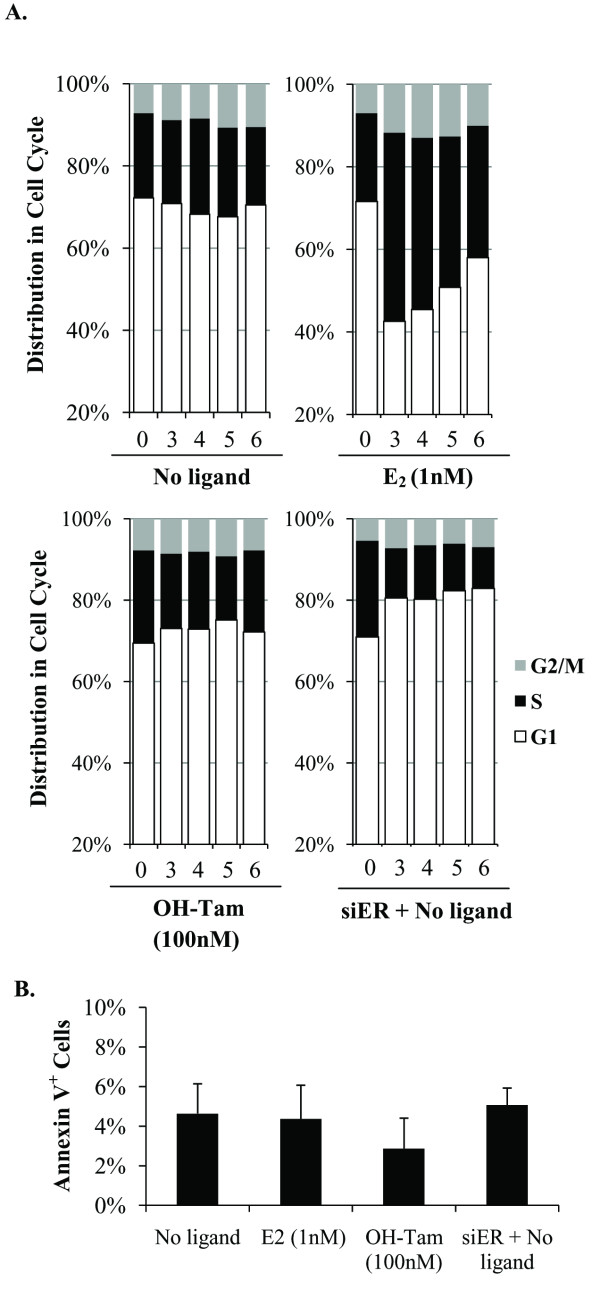
**Hormone-independent effects of ER on cell cycle phase distribution in hormone-sensitive MCF-7 cells**. Hormone-depleted MCF-7 cells were transfected with either ER siRNA or control siRNA and maintained in hormone-depleted conditioned media without further replenishment of the media. Twenty-four hours after transfection, cells were treated with vehicle (No ligand), E_2 _(1 nM) or OH-Tam (100 nM); the treatments were repeated every 48 hours without changing the media. **(A) **On the indicated days, the cells as treated above were harvested for flow cytometry analysis to determine their cell cycle phase distribution. **(B) **On Day 4 of the above treatment, cells were harvested to measure the proportion of apoptotic cells by Annexin V staining. *P*-values for the differences noted in the text were ≤0.001.

The net proliferation rate of MCF-7 cells under various conditions indicated in Figure 1 may be determined by changes in the rates of cell cycling as well as the rates of cell death. Figure 2A illustrates that during basal proliferation approximately 20% of the cells were in S-phase. Whereas E_2 _roughly doubled the proportion of S-phase cells, OH-Tam (100 nM) did not appreciably alter the basal cell cycle distribution (Figure 2A). However, knocking down ER in the hormone-depleted cells decreased the S-phase cells by approximately 50% (Figure 2A); the S-phase inhibition was accompanied by an increase in the proportion of cells in G1 phase indicating that the ER knockdown inhibited cell proliferation by inducing cell cycle arrest.

**Figure 3:**
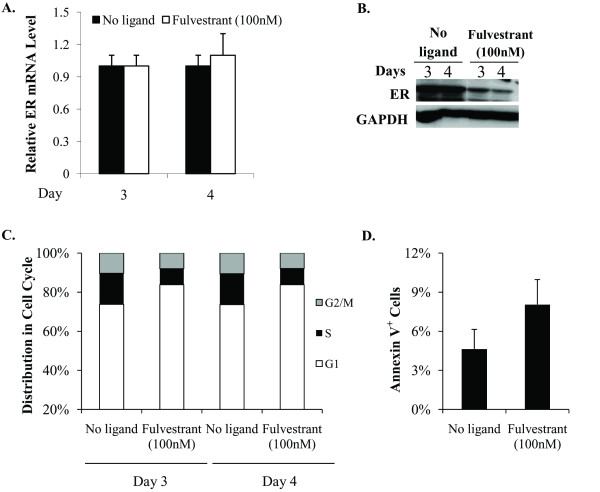
**Effect of fulvestrant on cell cycle phase distribution in hormone-depleted MCF-7 cells**. Hormone-depleted MCF-7 cells were treated with vehicle control or fulvestrant (100 nM) and maintained in hormone-depleted conditioned media without further replenishment of the media. The treatments were repeated every 48 hours for up to five days. **(A) **Cells were harvested after three and four days of treatment as described above and total RNA extracted from them. The mRNA for ER was measured by real time RT-PCR and the values were normalized to those for GAPDH. **(B) **Cells were harvested after three and four days of treatment as described above for western blot analysis using antibody to ER. GAPDH was probed as a loading control. **(C) **The cells treated as above were harvested on days 3 and 4 for flow cytometry analysis to determine their cell cycle phase distribution. (D) On Day 4 of the above treatment, cells were harvested to measure the proportion of apoptotic cells by Annexin V staining. *P*-values for the differences noted in the text were ≤0.001.

As an alternative method of depleting ER, the hormone depleted MCF-7 cells were treated with fulvestrant, a well established SERD, which causes proteolytic degradation of ER. As expected, fulvestrant treatment resulted in a substantial decrease in ER protein (Figure 3B), without affecting the level of ER mRNA (Figure 3A). Similar to knocking down ER with siRNA, treatment with fulvestrant caused cell cycle arrest (Figure 3C), providing complementary evidence for the role of apo-ER in supporting cell cycling in hormone depleted MCF-7 cells.

In contrast to cell cycle distribution the rate of apoptosis, measured by staining the cells for Annexin V did not show a significant change due to ligand treatment or knocking down ER compared to hormone depletion (Figure 2B). Fulvestrant treatment modestly increased the proportion of Annexin V positive cells (from 4.6% to 8%) (Figure 3D). Therefore, the principal mechanism by which ER supports basal proliferation of hormone-sensitive MCF-7 cells is by promoting cell cycle progression.

### Apo-ER supports basal cycling of MCF-7 cells through regulation of apo-RARα

**Figure 4:**
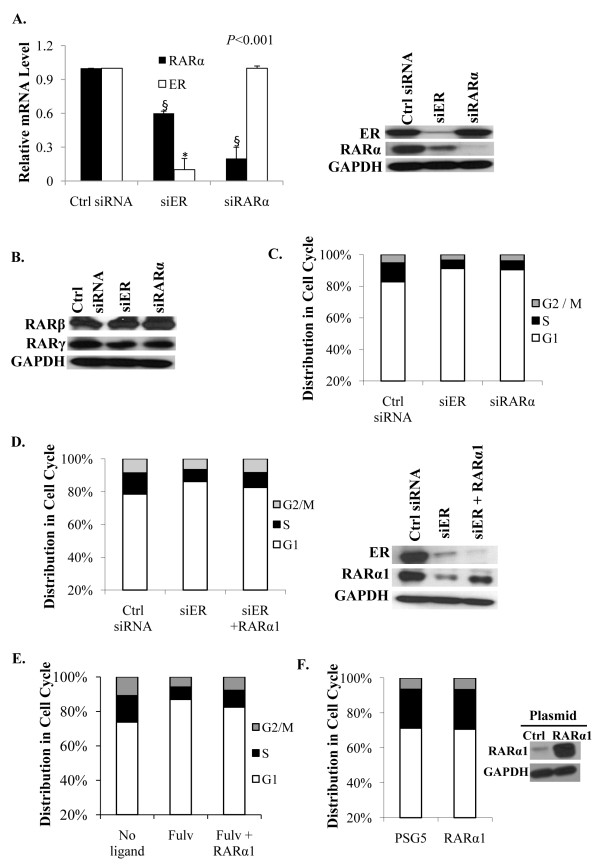
**Role of RARα in mediating the hormone-independent effect of ER on basal level cell cycling**. **(A) **Effect of knocking down either ER or RARα on the mRNA levels (left panel) and protein levels (right panel) of ER and RARα. Cells were transfected with control siRNA, ER siRNA or RARα siRNA and four days later the cells were harvested to extract total RNA for the measurement of ER and RARα mRNA by real time RT-PCR; the values were normalized those for GAPDH (control). The cells were also harvested four days after transfection for western blot analysis using antibody to either ER or RARα; the blots were probed for GAPDH as a loading control. **(B) **The cells were transfected as described for Panel A and the cell lysates were probed by western blot using antibodies specific for RARβ and RARγ; GAPDH was probed as a loading control. **(C) **Cells transfected as described in Panel A with control siRNA, ER siRNA and RARα siRNA were analysed by flow cytometry for cell cycle phase distribution. **(D) **RARα1 expression plasmid and siRNA against ER were co-transfected into hormone-depleted MCF-7 cells by nucleofection. As controls, cells were co-transfected with either control siRNA or ER siRNA and the vector plasmid. Cells were harvested three days after transfection and the cell cycle phase distribution determined by flow cytometry (left panel). The cells were also harvested at the same time for western blot analysis of the lysates using antibody to ER and RARα (right panel); GAPDH was probed as a loading control. **(E) **RARα1 expression plasmid or control vector plasmid was transfected into hormone-depleted MCF-7 cells using Fugene 6. The cells were treated with fulvestrant (100 nM) or vehicle and harvested after 72 h. The cell cycle phase distribution was determined by flow cytometry. **(F) **The cells were transfected as described in Panel E and harvested 96 hours after transfection. The cell cycle phase distribution was determined by flow cytometry (left panel) and the cell lysates were probed by Western blot using antibody specific for RARα (right panel); GAPDH was probed as a loading control.

Among the list of genes whose expression decreased significantly upon knocking down ER in MCF-7 cells depleted of hormone (Additional files 1, 2, 3, 4 and 5), we observed a decrease in RARα. Since the actions of antiestrogens and retinoids on breast cancer cells are profoundly regulated by an ER-RAR axis, it was of interest to examine a possible functional relationship between the two receptors in the absence of ligand in the context of basal cycling of ER+ breast cancer cells. In hormone depleted MCF-7 cells, knocking down ER decreased the level of RARα mRNA by about 40% and to a greater extent the RARα protein (Figure 4A). However, the basal ER level was unaltered by knocking down RARα (Figure 4A). Knocking down either ER or RARα did not significantly alter the expression of RARs -β and -γ (Figure 4B). The results indicate that apo-RARα is specifically regulated by apo-ER but not vice versa.

Similar to knocking down ER, knocking down RARα in hormone-depleted MCF-7 cells decreased the fraction of S-phase cells (Figure 4C). To test whether the effect of knocking down ER on the basal cycling of MCF-7 cells could be mediated by apo-RARα, the latter protein was introduced ectopically at the time of knocking down ER by co-transfecting an RARα isoform1 expression plasmid (Figure 4D). Restoring RARα at a level approaching the original basal level of the protein partially rescued cell cycling (Figure 4D). Similarly, RARα also partially rescued basal cell cycling in MCF-7 cells treated with fulvestrant (Figure 4E). On the other hand, overexpression of RARα1 had no impact on the cell cycle distribution, indicating that the endogenous level of RARα1 is optimal for supporting the basal level of cell cycling (Figure 4F). Taken together, the above results demonstrate that the ability of apo-ER to support basal cell proliferation is mediated to a large extent by its ability to support the expression of the basal level of apo-RARα.

### Apo-ER and apo-RARα support cell cycle in MCF-7 cells without selectivity with respect to ErbB2 and ErbB3 status

**Figure 5:**
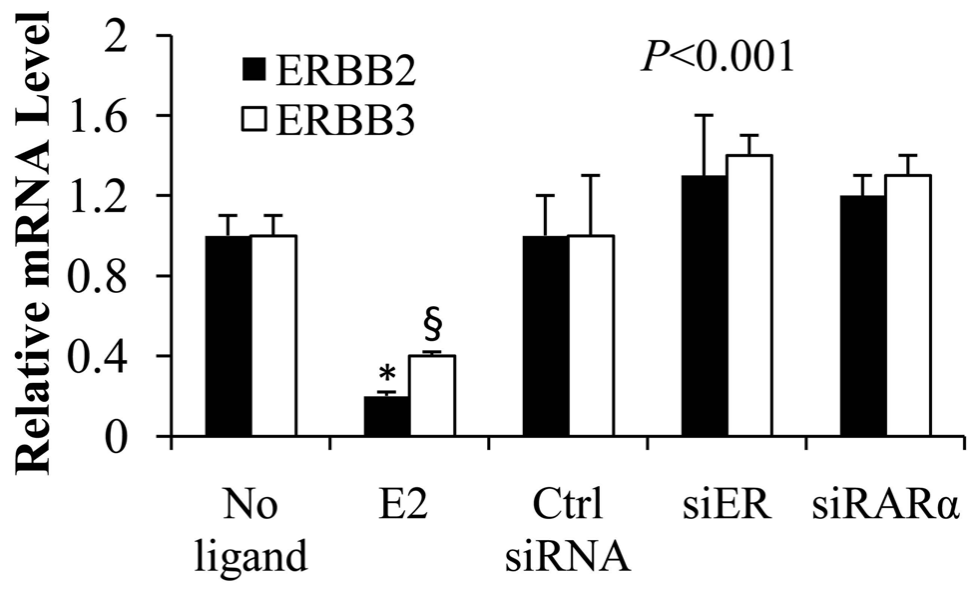
**ERBB2 and ERBB3 mRNA levels following depletion of apo-ER or apo-RARα1 in MCF-7 cells**. Cells were treated with vehicle, E2 (1 nM) or transfected with control siRNA, ER siRNA or RARα siRNA. Ninety-six days later the cells were harvested to extract total RNA for the measurement of ERBB2 and ERBB3 by real time RT-PCR. Values are normalized to the GAPDH values. *P*-values for the differences noted in the text were ≤0.001.

Since the MCF-7 cell line comprises a heterogeneous population of cells, and since ErbB2 and ErbB3 overexpression is associated with resistance to hormonal adjuvants it was of interest to test whether the ErbB2 and ErbB3 status of the cells was related to the dependence of basal cell cycling of MCF-7 cells on apo-ER or apo-RARα. As expected, treating hormone depleted MCF-7 cells with E_2 _inhibited ErbB2 and ErbB3 mRNA expression (Figure 5). Following knockdown of either ER or RARα, there was not a significant change in the mRNA levels of ErbB2 and ErbB3 compared to the hormone depleted control cells (Figure 5). Thus, the regulation of cell cycling of MCF-7 cells by apo-ER or apo-RARα did not show an obvious selectivity for a subpopulation of cells distinguishable by their ErbB2 or ErbB3 status.

### MCF-7 cells and several clinical breast tumors express isoform 1 of RARα

**Figure 6:**
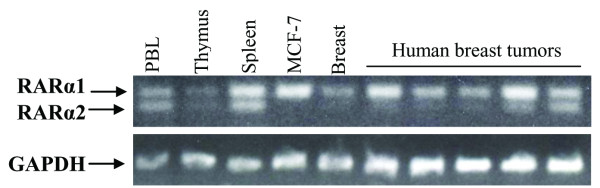
**Identification of major RARα isoforms in various normal tissues and in breast cancer**. Total RNA was extracted from MCF-7 cells, five human breast tumors and normal tissue controls (peripheral blood leukocytes, thymus, spleen and breast) and reverse transcribed into cDNA by RT-PCR. The cDNA fragments were amplified by competitive PCR using forward primers specifically against RARα1 and RARα2 and a common reverse primer. The PCR products were identified by electrophoresis on a 2% agarose gel by ethedium bromide staining. The cDNA for GAPDH was amplified in each sample as an internal control.

Given the significant differences in structure and regulation between the two RARα isoforms [34] it was of interest to determine whether only one or both isoforms were relevant in the regulation of RARα by apo-ER in breast cancer cells. cDNA prepared from the total RNA of MCF-7 cells as well as five breast tumors and normal human tissue controls (breast, spleen, thymus and peripheral blood leukocytes) were analyzed for RARα isoform expression by PCR. As seen in Figure 6, whereas both RARα1 and RARα2 were expressed in spleen, thymus and peripheral blood leukocytes, normal breast and MCF-7 cells as well as four of the five breast tumors expressed virtually exclusively RARα1 (Figure 6). The observations made in the limited number of clinical tumors need to be extended to a larger number of samples; nevertheless, the observation is consistent with previous evidence [35] for epigenetic silencing of RARα2 expression in MCF-7 cells and, together with the preceding observation that RARα1 rescued basal cell cycling in the ER knockdown cells, indicates that RARα1 is the relevant receptor isoform in the current study of breast tumor cells.

### Apo-RARα1 mediates regulation by apo-ER of tamoxifen-insensitive gene complements principally engaged in the cell division cycle

**Figure 7:**
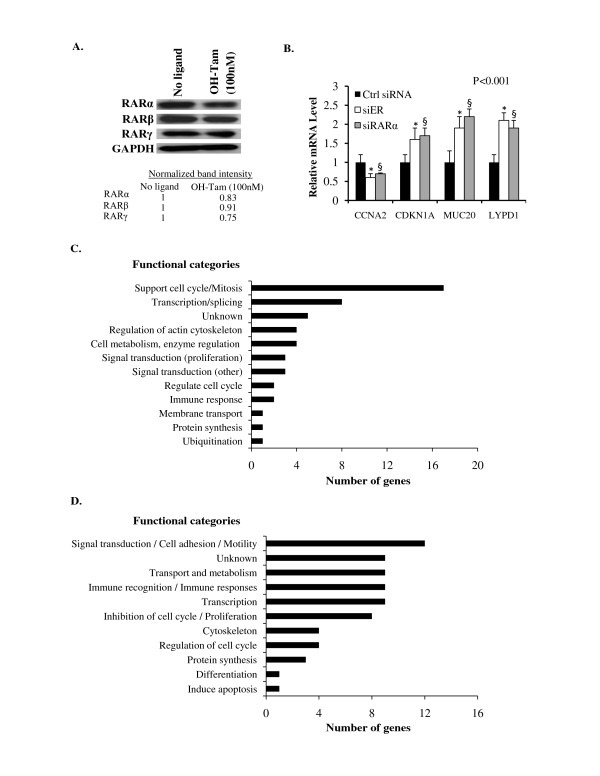
**Tamoxifen-insensitivity of RAR expression and the functional categories of target genes of the apo-ER apo-RARα1 axis**. **(A) **Hormone-depleted MCF-7 cells were treated with OH-Tam (100 nM) or vehicle control. Seventy-two hours later, the cell lysates were prepared and analysed by western blot using antibodies specific for RARα, RARβ or RARγ; GAPDH was probed as a loading control. The band intensities are normalized to GAPDH and equalized to a value of 1 for the untreated sample. **(B) **Real time RT-PCR analysis to confirm the effect of knocking down either ER or RARα on the mRNA levels of representative target genes found by Affymetrix DNA microarray in this study to be regulated by apo-ER and apo-RAR in MCF-7 cells: Cells were transfected with control siRNA, ER siRNA or RARα siRNA and four days later the cells were harvested to extract total RNA for the measurement of the relevant mRNAs; the values were normalized those for GAPDH (control). The *P*-values for the differences noted in the text were <0.001. **(C) **and **(D) **mRNA profiling was performed to identify common target genes of apo-ER and apo-RARα1 in MCF-7 cells. Apo-ER and apo-RARα1 were knocked down separately in hormone-depleted MCF-7 cells. Seventy-two hours after transfection with the appropriate siRNA, total mRNA was extracted and mRNA profiling was carried out using Affymetrixs microarray analysis. *P*-values for the differences noted in the text were ≤0.001.

In contrast to the effect of knocking down ER, the basal level of RARα1 did not appreciably decrease due to tamoxifen treatment (Figure 7A); the basal levels of RARs -β and -γ also only decreased by a relatively small extent due to tamoxifen treatment (Figure 7A). The gene targets downstream of the apo-ER → apo-RARα1 pathway were identified by mRNA profiling using Affymetrix microarray analysis. Accordingly, data from separate ER knockdown and RARα1 knockdown experiments in hormone depleted MCF-7 cells were used to identify overlapping sets of genes that were either up or down regulated by both ER and RARα1. The Affymetrix microarray analysis was validated for a few target genes known to regulate cell proliferation by real time RT-PCR; they include CDKN1A, MUC20 and LYPD1, which are negative regulators of cell proliferation whose basal expression was repressed by apo-ER and apo-RAR as well as CCNA2, a positive regulator of proliferation whose basal expression was supported by apo-ER and RARα1 (Figure 7B).

Table 1 lists the common target genes of apo-ER and apo-RARα1 whose basal expression was supported by both apo-ER and apo-RARα1 in a tamoxifen-insensitive manner; these genes were identified based on a decrease in basal mRNA level by at least 40% due to transfection of cells with siRNA specific for either ER or RARα1. Of the 53 annotated genes that were identified in this manner, gene ontology analysis (DAVID Bioinformatics Resources 2008) [36,37] revealed genes known to support the cell division cycle and mitosis as the predominant functional category (Figure 7C); the gene sets included additional functional categories that support proliferation (Figure 7C).

Table 2 lists the common target genes of apo-ER and apo-RARα1 whose basal expression was decreased by apo-ER or apo-RARα1 in a tamoxifen-insensitive manner; these genes were identified based on an increase in basal mRNA level by at least 50 percent due to transfection of cells with siRNA specific for either ER or RARα1. Of the 68 annotated genes that were identified in this manner, gene ontology analysis (DAVID Bioinformatics Resources 2008) revealed several categories of genes (Figure 7D); however, among genes functionally related to proliferation, there was enrichment for those that are known to negatively regulate cell proliferation (Figure 7D) in contrast to the genes activated by apo-ER/apo-RARα1 noted above.

### The common gene targets of apo-ER and apo-RARα1 are generally insensitive to ATRA but are enriched in RAR binding sites in associated chromatin regions

In the classical mechanism of the transcriptional activity of class II nuclear receptors, including RAR, the apo-protein is in a repressive association with the target gene and the binding of agonist to the receptor results in gene activation due to a co-regulator switch [38]. However, Affymetrix microarray analysis of MCF-7 cells following treatment with ATRA (1 μM, 24 h) indicated that only a fraction of the genes regulated by apo-ER/apo-RARα1 were regulated by ATRA (Tables 1 and 2). Specifically, among the 53 genes whose basal expression was supported by apo-ER/apo-RARα1, 6 genes were inhibited by ATRA to <50 percent and 1 was activated >2-fold (Table 1). On the other hand, among the 68 genes whose basal expression was repressed by apo-ER/apo-RARα1, 15 genes were activated by ATRA >2-fold and 10 genes were inhibited to <50 percent. Notably, none of the ATRA regulated genes in Tables 1 and 2 are functionally associated with the cell division cycle.

Since putative RAR binding sites have been globally mapped in the chromatin of MCF-7 cells [39], we used this information to identify the presence of RAR binding sites associated with the apo-ER/apo-RARα1 target genes listed in Tables 1 and 2. As indicated in the Tables, 18 of the 53 genes in Table 1 and 14 of the 68 genes in Table 2 were associated with RAR binding sites within a distance of 10 kb of the transcription start sites. The genes in Table 1 had significant enrichment (Hypergeometric test) for RAR binding sites within 10 kb with *P *= 0.03. The enrichment for RAR binding sites for the genes in Table 2 had a *P*-value of 0.19. Thus, despite the low frequency with which the apo-ER/apo-RARα1 target genes are regulated by ATRA, there is a significant enrichment among them for associated RAR binding sites, largely among gene targets that are insensitive to ATRA.

### Down-regulation of RARα1 inhibits hormone-independent cell cycle progression in other breast cancer cell lines

**Figure 8:**
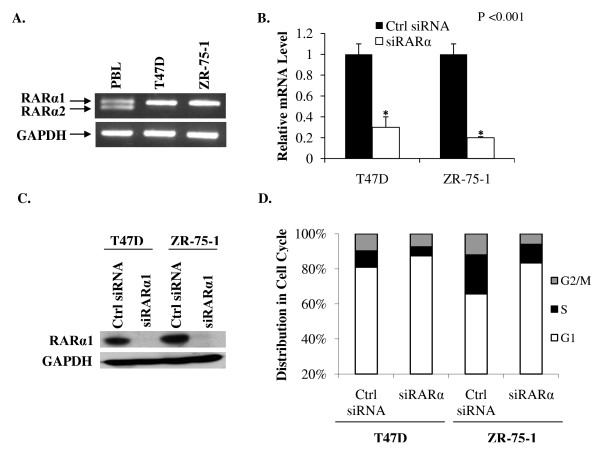
**Effect of depleting RARα1 on cell cycle phase distribution of hormone-depleted T47 D and ZR-75-1 cells**. **(A) **Total RNA was extracted from T47 D and ZR-75-1 cells and normal tissue controls (peripheral blood leukocytes, thymus and spleen) and reverse transcribed into cDNA by RT-PCR. The cDNA fragments were amplified by competitive PCR using forward primers specifically against RARα1 and RARα2 and a common reverse primer. The PCR products were identified as previously described (Figure 5). The cDNA for GAPDH was amplified in each sample as an internal control. In **B **and **C**, cells were transfected with control siRNA or RARα siRNA and 72 hours later the cells were harvested to extract total RNA or to prepare cell lysates. In B, the mRNA for RARα1 was measured by real time RT-PCR and the values were normalized those for GAPDH (control). In C, the cells lysates were analyzed by western blot using antibody to RARα; the blots were probed for GAPDH as a loading control. (D) Seventy-two hours after transfection with control siRNA or RARα siRNA, T47 D and ZR-75-1 cells were harvested for flow cytometry analysis to determine their cell cycle phase distribution. *P*-values for the differences noted in the text were ≤0.001.

Since down-regulating, rather than activating RARα1 may be an attractive therapeutic strategy in concert with estrogen ablation in breast cancer, it was of interest to test the effect of depleting RARα1 in other model cell lines. Similar to MCF7 cells, RARα1 but not RARα2 was expressed in the estrogen-sensitive T47 D and ZR-75-1 cell lines as determined by competitive RT-PCR (Figure 8A). Interestingly, in contrast to MCF-7 cells, RARα1 was not regulated by apo-ER and further knocking down apo-ER did not affect cell cycling (data not shown). Thus, the regulation of RARα1 by apo-ER is somehow disrupted in these cells; this finding further supports the conclusion that regulation of RARα1 underlies the effect of apo-ER on cell cycling. Nevertheless, in either cell line, knocking down RARα1 (Figure 8B showing RARα1 mRNA and 8C showing RARα1 protein) inhibited cell cycle progression in the absence of hormone (Figure 8D), similar to MCF7 cells. The results demonstrate a consistent role for RARα1 in supporting a basal level cycling in hormone depleted breast cancer cells.

## Discussion

ER is known to regulate genes in a ligand-independent manner [40,41]. Hormone-independent actions of ER play an important role in supporting the growth of hormone-refractory breast tumors [12]. On the other hand, studies of gene regulation by ER in estrogen-sensitive breast cancer cells have mostly focused on estrogen-responsive genes that have profound roles in tumor growth and development and the effects of tamoxifen on gene regulation by estrogen [42]. The findings of this study, however, highlight a potentially significant mechanism of hormone-independent transcriptional action of ER in hormone-sensitive breast cancer cells. This action of ER is clearly a major contributor to the ability of hormone-sensitive breast cancer cells to maintain a basal level of proliferation under conditions of hormone-depletion. This effect of apo-ER occurred primarily through supporting the cell division cycle. Remarkably, the action of apo-ER was also rather insensitive to tamoxifen at a dose that is clinically relevant to circulating concentrations of the drug that induce all of the surrogate biomarkers of clinical response [43,44]. Similar to clinical breast tumors, breast cancer cell lines are heterogeneous and can yield clonal populations of inherently tamoxifen resistant cells that are variably ER-dependent [45]; nevertheless, the fraction of S-phase cells in hormone-depleted or tamoxifen-treated cells under the *in vitro *conditions in this study was much higher than the frequency of emergence of aggressively growing colonies in tamoxifen-treated cultures [45]. Therefore, it is likely that the basal level of S-phase cells observed in hormone-depleted or tamoxifen treated cultures represent a substantial proportion of cells in which the cell cycle progression is slowed. In a tumor environment, however, this slow proliferation must be offset by cell death, resulting in an overall tumoristatic effect. Since a basal level of cell division is an essential pre-condition for progressive events leading to the eventual development of resistance of breast tumors to hormonal adjuvant therapy, understanding the mechanism of the hormone-independent effects of ER in hormone-sensitive cells is important.

In hormone-sensitive breast cancer cells, the well-established ER-RAR axis has been best characterized in the context of ligand effects (estrogen, retinoids, tamoxifen + retinoids) [20,46]. The results of this study however establish that in hormone-sensitive cells that are depleted of hormone or treated with tamoxifen, a major mechanism by which ER supports the cell cycle is by supporting the basal expression of RARα1. The role of RARα1 in mediating the action of apo-ER is strongly evident from the following observations: (i) In hormone-depleted cells, apo-ER maintained the basal expression level of RARα1 but was not itself regulated by RARα1; (ii) The regulation of RARα1 by apo-ER was insensitive to tamoxifen; (iii) Knocking down RARα1 negatively impacted the basal cell cycle progression and restoring basal apo-RARα1 levels rescued basal level cell division following depletion of ER; (iv) Apo-RARα1 independently regulated a complement of genes in a manner that strongly favored cell division similar to their regulation by apo-ER. This mechanism was remarkable for the following reasons. First, apo-ER regulated the α1 subtype of RAR but not RARs -β or - γ. Second, most of the common target genes of apo-ER and apo-RARα1 including all of the genes involved in the cell division cycle were insensitive to ATRA. These findings suggest that a major molecular mechanism by which apo-ER supports basal cell division in hormone-sensitive breast cancer cells may not be sensitive to conventional RAR ligands (agonists), but would be predictably opposed by specific inactivators or down-regulators of RARα1.

The cell cycle regulation, which occurs through the apo-ER/apo-RARα1 axis, could theoretically exclude a subpopulation(s) of cells; such a subpopulation(s) could represent tumor cells that are either inherently resistant to hormonal adjuvants or that undergo progressive changes leading to resistance. Whereas the findings in this study do not preclude this possibility, we found no evidence for residual cycling cells following depletion of apo-ER or apo-RARα1 that were characterized by ErbB2 or ErbB3 overexpression, common features associated with a resistant phenotype [47,48].

It is well established that the RARα gene is activated by estrogen; however, there is evidence in the literature that ER associates at a basal level with the core promoter of the RARα gene by tethering to DNA bound Sp1 [49]. Apo-ER may thus directly regulate the RARα gene to maintain the basal expression level of RARα1. The observation that the RARα1 protein level decreased more dramatically than its mRNA upon knocking down ER suggests that apo-ER also regulates RARα1 by additional posttranscriptional mechanisms.

The results of this study further indicate that multiple molecular mechanisms must underlie the downstream action of apo-RARα1 on target genes in the context of mediating the effects of apo-ER. The apo-ER/apo-RARα1 axis regulates genes in both a positive and a negative manner to support cell division; both sets of target genes were enriched for associated chromatin sites of RAR binding, suggesting that RARα1 must act on these target genes by direct as well as indirect mechanisms. RAR belongs to the Class II subfamily of nuclear receptors, which typically, in their ligand-free (apoprotein) form, maintain a transcriptionally repressed state of target genes activated by the corresponding agonists [38]. However, only a small fraction of genes regulated by the apo-ER-RARα1 axis appeared to be regulated by this classical mechanism of action of RARα1, since (i) the genes repressed by apo-RARα1 were largely insensitive to ATRA and (ii) most genes activated by apo-RARα1 were ATRA-insensitive. Therefore, apo-RARα1 must act by non-classical mechanisms on most of the target genes, including those with associated RAR binding sites.

RARα is consistently present in the nucleus in breast tumors and its expression levels correlate with that of the proliferation marker, ki-67 [50]. The functional RARα isoform in different hormone-sensitive breast cancer cell lines and that identified in a limited number of breast tumors was almost exclusively of type 1, an isoform that is believed to be genetically redundant [51]. The findings reported here would predict that agents that selectively target the α1 subtype of RAR for functional inhibition or degradation would significantly enhance hormone ablation therapy in breast cancer since this would further decrease cycling cells. Since the effect of depleting RARα1 occurs through a different gene regulatory program compared with retinoid agonists, and since RARα1 is genetically redundant and could be the major or only RARα subtype in breast cancer cells, this new approach (rather than the use of RAR agonists) to enhancing hormonal adjuvant therapy in breast cancer may be clinically more acceptable. The structural divergence of the two RARα isoforms arising from alternative promoter usage and alternative splicing includes differences in functional sub-domains [34] which may enable their differential targeting with pharmacological agents. This approach may have fewer side effects than SERDs due to a redundancy of RAR subtypes in other tissues. Studies are underway to test this concept in pre-clinical models of hormone-sensitive breast cancer.

## Conclusions

We have observed that in hormone-sensitive breast cancer cells, there is a hormone-independent component through which ER supports proliferation and that apo-RARα1 is a major mediator of this effect. The data also show that the majority of genes regulated by apo-RARα1 do not confirm to the classical model of gene regulation by Class II nuclear receptors since they are not regulated by ATRA. Further, selectively down-regulating RARα1 might significantly enhance hormone ablation therapy in breast cancer since this would further decrease cycling cells. Since the effect of depleting RARα1 occurs through a different gene regulatory program compared with retinoid agonists, and since RARα1 is genetically redundant and appears to be the only RARα subtype in breast tumor cell lines and possibly the major subtype in clinical tumors, this new approach (rather than the use of RAR agonists) to enhancing hormonal adjuvant therapy in breast cancer may be clinically more acceptable. RARα1 is also structurally divergent from RARα2, which is expressed in normal tissues, so that it should be possible to develop selective down-regulators of RARα1.

## Abbreviations

AI: aromatase inhibitors; ATRA: all-*trans *retinoic acid; DMEM: Dulbecco's minimum essential medium; FBS: fetal bovine serum; ER: estrogen receptor α; GAPDH: glyceraldehyde-3- phosphate dehydrogenase; GFP: Green Fluorescent Protein; RAR: retinoic acid receptor; OH-Tam: 4-hydroxytamoxifen; SERM: selective estrogen receptor modulator; SERD: selective estrogen receptor downregulator.

## Competing interests

The authors declare that they have no competing interests.

## Authors' contributions

MDS conducted most of the experiments and contributed to the planning and analysis. MR, MP and IK contributed to the execution of some of the experiments. RT contributed to the bioinformatics analysis. IM contributed with an intellectual clinical perspective to the studies. MR was responsible for the overall direction of the project.

## Supplementary Material

Additional file 1 **Microarray - Control sample**. Affymetrix DNA microarray analysis of MCF-7 cells transfected with non-targeting siRNA control and treated with vehicle. Click here for file

Additional file 2 **Microarray - OH-Tam treatment**. Affymetrix DNA microarray analysis of MCF-7 cells transfected with non-targeting siRNA control and treated with OH-Tam. Click here for file

Additional file 3 **Microarray - ER knockdown**. Affymetrix DNA microarray analysis of MCF-7 cells transfected with siER and treated with vehicle. Click here for file

Additional file 4 **Microarray - Control sample 2**. Affymetrix DNA microarray analysis of MCF-7 cells transfected with non-targeting siRNA control. Click here for file

Additional file 5 **Microarray - RARα knockdown**. Affymetrix DNA microarray analysis of MCF-7 cells transfected with siRARα. Click here for file
